# HLA-DQA1 and HLA-DQB1 Alleles, Conferring Susceptibility to Celiac Disease and Type 1 Diabetes, Are More Expressed Than Non-Predisposing Alleles and Are Coordinately Regulated

**DOI:** 10.3390/cells8070751

**Published:** 2019-07-19

**Authors:** Federica Farina, Stefania Picascia, Laura Pisapia, Pasquale Barba, Serena Vitale, Adriana Franzese, Enza Mozzillo, Carmen Gianfrani, Giovanna Del Pozzo G

**Affiliations:** 1 Institute of Genetics and Biophysics “Adriano Buzzati Traverso”-CNR, 80131 Naples, Italy; 2 Institute of Biochemistry and Cell Biology-CNR, 80131 Naples, Italy; 3 Department of Translational Medical Science (DISMET), Section of Pediatrics, University of Naples Federico II, 80131 Naples, Italy

**Keywords:** autoimmunity, risk genes, expression, regulation

## Abstract

HLA DQA1*05 and DQB1*02 alleles encoding the DQ2.5 molecule and HLA DQA1*03 and DQB1*03 alleles encoding DQ8 molecules are strongly associated with celiac disease (CD) and type 1 diabetes (T1D), two common autoimmune diseases (AD). We previously demonstrated that DQ2.5 genes showed a higher expression with respect to non-CD associated alleles in heterozygous DQ2.5 positive (HLA DR1/DR3) antigen presenting cells (APC) of CD patients. This differential expression affected the level of the encoded DQ2.5 molecules on the APC surface and established the strength of gluten-specific CD4^+^ T cells response. Here, we expanded the expression analysis of risk alleles in patients affected by T1D or by T1D and CD comorbidity. In agreement with previous findings, we found that DQ2.5 and DQ8 risk alleles are more expressed than non-associated alleles also in T1D patients and favor the self-antigen presentation. To investigate the mechanism causing the high expression of risk alleles, we focused on HLA DQA1*05 and DQB1*02 alleles and, by ectopic expression of a single mRNA, we modified the quantitative equilibrium among the two transcripts. After transfection of DR7/DR14 B-LCL with HLA-DQA1*05 cDNA, we observed an overexpression of the endogenous DQB1*02 allele. The DQ2.5 heterodimer synthesized was functional and able to present gluten antigens to cognate CD4^+^ T cells. Our results indicated that the high expression of alpha and beta transcripts, encoding for the DQ2.5 heterodimeric molecules, was strictly coordinated by a mechanism acting at a transcriptional level. These findings suggested that, in addition to the predisposing HLA-DQ genotype, also the expression of risk alleles contributed to the establishment of autoimmunity.

## 1. Introduction

The human leukocyte antigen (HLA) class II heterodimeric molecules, composed by the alpha and beta chains, are encoded by many different alleles, which generate the high polymorphism characteristic of this locus. The genes encoding DR and DQ isotypes are the main risk factors associated with several autoimmune diseases. Type 1 diabetes (T1D) and celiac disease (CD) are autoimmune disorders, affecting between 0.5% and 1% of the general population. Very frequently, these two diseases co-occur in families, and approximately 4–9% of patients with T1D also have CD, while patients with CD are at increased risk of developing T1D [1]. CD and T1D share immunopathogenic mechanisms, although the autoreactive T cells and autoantibodies are directed against different autoantigens, such as insulin, GADA65, and IA-2 in T1D [2] and tissue transglutaminase in CD [3]. The HLA-DRB1, HLA-DQA1, and HLA-DQB1 genes display a major component of familial clustering in both T1D and CD. Subjects at high risk to develop T1D carry either DQ2.5 haplotype, encoded by DQA1*05:01 and DQB1*02:01 or DQ8 haplotype, encoded by DQA1*03:01 and DQB1*03:02 [4]., whereas the most prominent association of CD is with HLA-DQ2.5 molecules [5]. The alleles encoding DQ2.5 molecule are in linkage disequilibrium (LD) with HLA-DRB1*03, while the DQ8 alleles are in LD with HLA-DRB1*04. More specifically, considering the subtype specificity, the DRB1*03:01 is found coupled to DQA1*05:01 and DQB1*02:01 almost exclusively, thus creating the haplotype called “DR3”, while DRB1*04:01 is found in LD with DQA1*03:01 but can have either DQB1*03:01 or DQB1*03:02 included in the haplotype. Both are referred to as “DR4” haplotypes, although that carrying the DQB1*03:02 allele (DQ8 haplotype) predisposes to disease and that with DQB1*03:01 allele (DQ7 haplotype) is protective [4,5]. T1D risk depends not only on the haplotypic context but also on the genotypic assets of the risk alleles. In fact, the highest risk is conferred by DR3/DR4 heterozygous genotype. The contribution of DR3/DR4 is higher than the sum of the individual DR3 and DR4 haplotypes. This group comprised T1D subjects carrying DRB1*03:01-DQA1*05:01-DQB1*02 on one chromosome and DRB1*04:01-DQA1*03:01-DQB1*03:02 on the other chromosome. One hypothesis for the increased risk is the putative presence of DQ heterodimers encoded by alleles in *trans*, in addition to the DQ molecules encoded by alleles in *cis*, on the cell surface of immune cells [6,7].

We have previously demonstrated [8] that APCs from celiac patients, carrying the DR3-DQ2.5 haplotype in homozygosis (DR3/DR3) or in heterozygosis (DR1/DR3) have a comparable ability to stimulate the CD4^+^ T cell activation and proliferation when challenged with an equivalent amount of gluten antigen. This finding is a consequence of the differential expression of DQA1*05 and DQB1*02 alleles compared to non-CD-associated ones, affecting the DQα1*05 and DQβ1*02 chain amount and the HLA-DQ2.5 surface heterodimers density. In the present work, we measured the expression of DQ2.5 and DQ8 risk genes in APC from patients affected by T1D and from patients affected by T1D and CD comorbidity, that could be relevant in the activation of antigen specific CD4 lymphocytes.

Previous papers from our lab have demonstrated the stoichiometric balance of two messengers encoding alpha and beta protein chains, by a mechanism coordinating the transcription and the mRNA processing. A ribonucleoprotein complex, binding the 5’UTR and 3′UTR of HLA class II transcripts, regulates the nucleus-cytoplasm export and degradation of these mRNA [9,10]. Moreover, it has been demonstrated that the ectopic over-expression of the DRB1 gene determines an increase of the endogenous DRA mRNA in non-professional APC, such as melanoma M14 cell line. In order to investigate the mechanism determining the high and the coordinated expression of DQA1*05 and DQB1*02 risk alleles, we have set up experiments aimed to perturb the stoichiometric balance of two mRNA and to assess the functionality of resulting heterodimers in the antigen presentation.

## 2. Materials and Methods

### 2.1. Patients Enrollment and Selection of Antigen Presenting Cells

Type 1 diabetes patients were enrolled at the Department of Pediatrics, University Hospital “Federico II” of Naples, where they are regularly followed up. Patients or their parents, in the case of children under 12 years old, were informed about the objective of the study and provided written informed consent in accordance with the ethical standards of the institutional, the national research committee, and in accordance with the 1964 Helsinki declaration and its later ethical amendments.

Peripheral blood mononuclear cells (PBMC) were isolated from blood samples of patients and healthy donors by Ficoll-Paque gradient separation. PBMC were used to prepare genomic DNA and total RNA. B lymphoblastoid cell lines (B-LCL), used as APCs, were obtained, as previously reported. All subjects enrolled were genotyped for DQA1 and DQB1 by using AllSet Gold SSP HLA-DQ Low Res kit (Thermo Fisher Scientific, Monza, Italy) and the genotypes are indicated in Table 1. In the functional test with T cells and in the nucleofection experiments we used DR1/DR3 positive B-LCL (B-LCL#5) from CD patient carrying DQA1*05-DQA1*01/DQB1*02-DQB1*05 genotype that was previously described [8] and DR7/DR14 positive B-LCL from a healthy donor carrying DQA1*02-DQA1*01/DQB1*02-DQB1*05 genotype.

### 2.2. Monoclonal Antibodies and Flow Cytometry Analysis

B-LCLs were harvested when they were at sub-confluence and were suspended at 10^6^ cells/mL in ice-cold PBS, 10% FCS and 1% NaN_3_. Then, 100 µl of cell suspension was plated in a 96 V-bottom plate and labeled with 10 μg/mL of primary or isotypes control monoclonal antibodies, or with 10μl of the hybridoma supernatant, previously titrated. The cells were incubated at 4 °C in the dark for 30 min, washed and thereafter labeled with secondary antibodies at a final concentration of 1 μg/mL in 3% BSA/PBS for an additional 30 min at 4 °C in the dark. The primary monoclonal antibodies used to reveal the cell surface HLA DQ expression were: SFR20-DQa5, a rat anti-HLADQA1*05 purified from a hybridoma supernatant, kindly provided by Prof Radka [11]; 2.12E11, a murine anti-HLA-DQB1*02, kindly provided by Prof L. Sollid [12]. Fluorochrome conjugated anti-rat IgG(-PE) and anti-mouse IgG (FITC) were used as secondary antibodies. All phenotypes were analyzed with FACSCanto II system and elaborated using the DIVA software (BD Biosciences, Milan, Italy).

### 2.3. cDNA Cloning and B-LCL Nucleofection

The full-length DQA1*05 and DQB1*02 cDNA were prepared using retrotranscribed RNA from B-LCL of homozygous DR3/DR3 positive CD patient (B-LCL#1), previously described [8]. By specific primers, we cloned both cDNA in pcDNA3 vector and named the constructs pDQA105 and pDQB102. Using pDQA105 as a template, we cloned the cDNA deleted of 3′UTR (pDQA105Δ). The correct cloning was assessed by sequencing (Eurofins, Munchen, Germany). We transfected DR1/DR3 B-LCL#5 with three constructs and DR7/DR14 B-LCL with pDQA105 by nucleofection using Amaxa Cell line Nucleofector kit V (Lonza, Euroclone, Milan Italy). Cells were harvested after 24 h for the RNA preparation and functional test and after 48 h for flow cytometry experiments.

### 2.4. RNA Quantization

Total RNA was prepared with the Aurum™ Total RNA kit (BIORAD, Milan, Italy), and 0.5 µg of RNA was used for reverse transcriptase reactions, performed using an iScript™ cDNA Synthesis kit (BIORAD). The number of specific transcripts was measured by qPCR using the Quanti Tect SYBR Green PCR Kit (BIORAD) through the DNA Engine Opticon Real-Time PCR Detection System (BIORAD). Each reaction was run in triplicates in the presence of 0.2 mM primers synthesized by Eurofins, and each experiment was performed four times. The primer sequences are reported in Table 2. The relative amount of specific transcripts was calculated by the comparative cycle threshold method [13] and β-actin transcript was used for normalization.

The newly synthesized RNA transcripts were captured by Click-iT Nascent RNA Capture Kit (ThermoFisher), according to manufacturer’s instructions. Briefly, B-LCLs, seeded at 50% confluency, were labeled with 0.2 mM ethynyl uridine (EU) and incubated at 37 °C for 16 h. Total RNA was prepared with TRIzol reagent (Life Technologies, Thermo Fisher Scientific, Monza, Italy). The EU-labeled RNAs were biotinylated with 0.5 mM biotin azide in Click-iT reaction buffer (Thermo Fisher Scientific, Monza, Italy). The biotinylated RNAs were precipitated and resuspended in distilled water. Purified RNA (0.5 µg) was bound to 25 μL of Dynabeads MyOne Streptavidin T1 magnetic beads in Click-iT RNA binding buffer. The RNA captured on the beads was used as template for cDNA synthesis. Reverse transcription was performed using the SuperScript VILO cDNA Synthesis Kit (Invitrogen, Thermo Fisher Scientific, Monza, Italy) following the manufacturer’s instructions. The number of specific transcripts was measured by qPCR using SsoAdvanced SYBR Green PCR Kit (BIORAD). The apparatus and primers used were the same as described above.

### 2.5. T Cell Functional Assay

A gliadin-reactive T cell line (TCL) was previously established from the jejunal biopsies of a DR3/DR3 homozygous CD patient in disease remission. The TCL was obtained by stimulating intestinal cells with irradiated autologous PBMC (1.5 × 10^6^) and gliadin peptide and expanded by cyclic stimulations with allogenic PBMC and phytohemagglutinin (PHA, 0.5 µg/mL) in complete medium (X-Vivo 15 medium supplemented with 5% AB-pooled human serum and antibiotics, Lonza). Cells were fed by adding IL-2 (50 UI/mL, R&D System) every 3 days. This TCL was reactive to DQ2.5-glia-γ1 peptide (PQQPQQSFPQQQQPA) [14]. When in resting phase, T cells (3 × 10^4^) were co-incubated with indicated B-LCL (1 × 10^5^) pulsed with DQ2.5-glia-γ1 peptide at concentration 0.1 and 1 µM. In some experiments B-LCL transfected with a pDQA105 construct or with pcDNA (empty vector) were used. Cell supernatants (50 µL) were collected after 48 h for the evaluation of INF-γ production, using standard sandwich ELISA procedure. The DQ2.5-glia-γ1 peptide was provided by CASLO ApS, (Kongens Lyngby, Denmark).

### 2.6. Statistical Analysis

All results are shown as the mean of at least three independent experiments. Statistical analysis was performed using the unpaired Student’s t-test with a two-tailed distribution. A *p*-value less than 0.05 was considered significant.

## 3. Results

### 3.1. The DQA1*03 and DQB1*03 mRNA Associated with T1D Risk Were More Abundant than the mRNA of Non-T1D Related Alleles

We genotyped the DNA of B-LCL and PBMC from patients affected by T1D alone or by T1D and CD comorbidity in order to identify the HLA-DQ genes (Table 1). The HLA-DR phenotype was designed by LD. We measured, by qPCR, the amount of DQA1*03, DQA1*05, DQB1*03, and DQB1*02 mRNA with respect to transcripts of alleles not associated with diseases. A value of 100% of mRNA amount was assigned when DQA1*05 (patients §3, §8), DQA1*03 (patients §18 and §20), DQB1*02 (patients §2, §3, §7, §8) and DQB1*03 (patients §18, §20) alleles were in homozygosis. For patients DQA1 and DQB1 heterozygous, the mRNA amount was expressed as a percentage of the total messenger of the gene.

**Figure 1:**
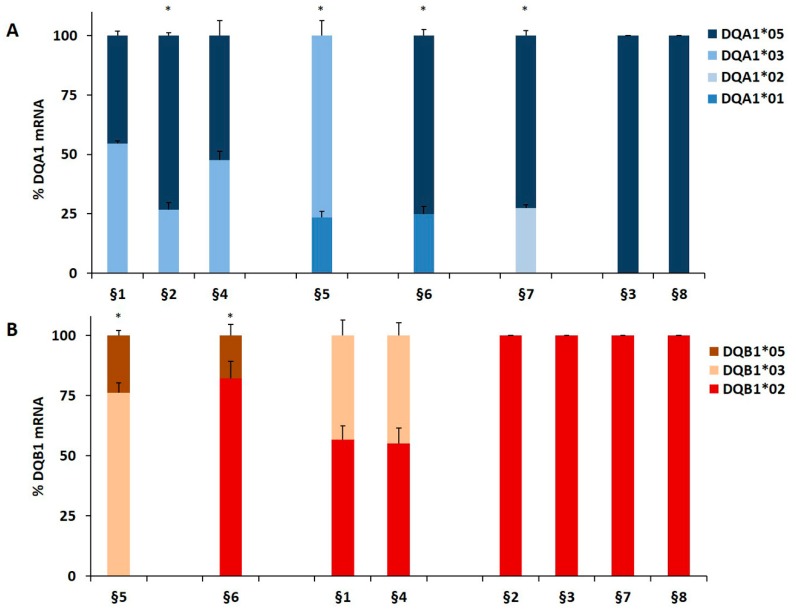

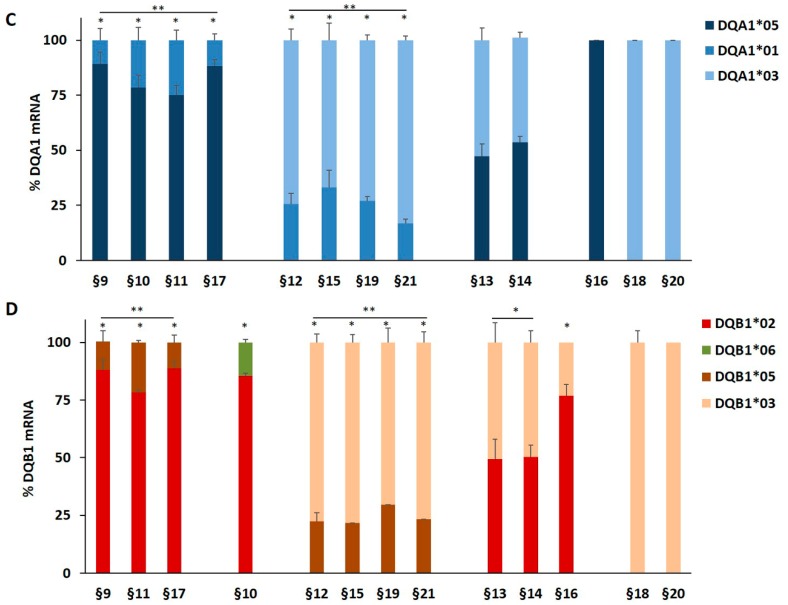
Expression of DQB1 and DQA1 genes. The gene expression is showed as percentages of the total DQA1 and DQB1 transcripts for each APC. Panel **A** and **C** show the expression of DQA1 alleles in B-LCL and PBMC, respectively; panel **B** and **D** show the expression of DQB1 alleles in B-LCL and PBMC, respectively. The APC are grouped by their genotypes; the significance, among the expression values of two alleles, was calculated for each patient and for the group with at least two samples (* *p* < 0.05, ** *p* < 0.005).

Our results demonstrated that in heterozygous B-LCL, the amount of DQA1*05 (patients §6, §7) and DQA1*03 (patient §5) mRNA was higher respect to DQA1*01 (patients §5, §6) and DQA1*02 (patient §7) mRNA, as showed in Figure 1A. Similar results were obtained in heterozygous PBMC, in which either DQA1*05 (patients §9, §10, §11, §17) and DQA1*03 (patients §12, §15, §19, §21) mRNA are more abundant than DQA1*01 transcript (Figure 1C). Moreover, either B-LCL (patients §1, §4, Figure 1A) and PBMC (patients §13, §14, Figure 1C) carrying DQ2.5/DQ8 genotypes showed a comparable amount of DQA1*03 and DQA1*05 mRNA, with exception of B-LCL §2 in which DQA1*05 was more abundant than DQA1*03 (Figure 1A). Similarly, we demonstrated that DQB1 risk alleles were more expressed with respect to the alleles non-T1D associated. The heterozygous B-LCL of patients §5 and §6, carrying DQB1*03 or DQB1*02, respectively, showed mRNA amount higher than DQB1*05 (Figure 1B), as well as the heterozygous PBMC, in which DQB1*02 allele (patients §9, §10, §11, §17) displayed a quantity of transcript greater than non-predisposing alleles (DQB1*05 and DQB1*06, Figure 1D). Finally, PBMC §12, §15, §19, and §21 (Figure 1D) expressed a higher level of DQB1*03 mRNA with respect to DQB1*05 mRNA, with the exception of PBMC §16 (Figure 1D) that showed a lower amount of DQB1*03 mRNA, in comparison to DQB1*02. Finally, the B-LCL of patients §1 and §4 (Figure 1B) and PBMC of patients §13 and §14, characterized by DQ2.5/DQ8 genotypes, showed a comparable amount of DQB1*03 and DQB1*02 mRNA (Figure 1D). We calculated the significance among the expression values of two alleles for each patient and for the group carrying the same alleles (Figure 1). The fold change (FC) of the expression value for each patient were more clearly reported in Table 3. Overall, these results demonstrated that disease predisposing alleles are, surprisingly, expressed at higher levels than non-associated alleles in all heterozygous APC. When DQA1*05 and DQB1*03 risk alleles, as well as DQB1*02 and DQB1*03, are in the same DQ2.5/DQ8 genotype, the cells expressed equal amounts of mRNA in all determinations done.

### 3.2. Analysis of Co-Regulated Expression of DQA1*05 and DQB1*02 mRNA

**Figure 2:**
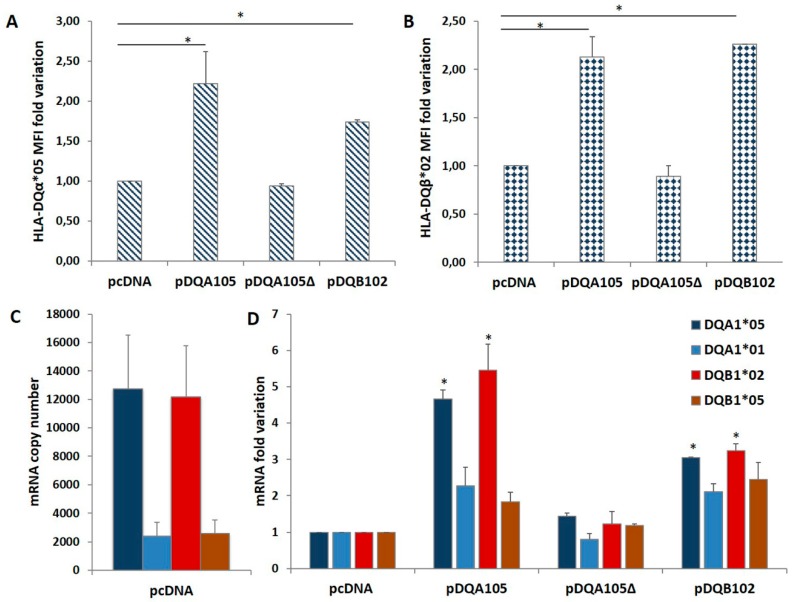
Coregulated expression of DQA1*05 and DQB1*02 CD risk alleles in DR1/DR3 B-LCL#5. This cell line was transfected with pcDNA, pDQA105, pDQA105Δ, and pDQB102 constructs. The surface expression of DQα1*05 (panel **A**) and DQ1β*02 (panel **B**) was measured by flow cytometry and reported as mean fluorescence intensity (MFI). The amount of DQA1*05 and DQB1*02 mRNA related to diseases, as well as the DQA1*01 and DQB1*05 mRNA, non-associated to pathologies, was reported as copy number in panel **C** and as fold variation in panel **D**. The *p*-value was calculated respect to cells transfected with empty vectors (* *p* < 0.05).

We investigated on the mechanism causing the differential expression of HLA genes associated with T1D and CD risk by nucleofection of the full-length DQA1*05 or DQB1*02 cDNA (pDQA105 and pDQB102) and the DQA1*05 deleted of 3′UTR (pDQA105Δ) cDNA in DR1/DR3 B-LCL#5. The aim was to verify if the ectopic expression of each alpha or beta mRNA affected the modulation of the other mRNA. 48 h after nucleofection with pDQA105 we observed an increased surface expression of DQα1*05 chain (Figure 2A), as expected, and an unexpected increment of DQ1β*02 chain (2,1 fold change of MFI) expressed by endogenous gene (Figure 2B). Similarly, when we transfected the B-LCL#5 with pDQB102, we observed a 2.3-fold increase of DQ1β*02 MFI (Figure 2B) and, 1.7-fold increment of DQα1*05 chain (Figure 2A). No variation in the MFI of DQα1*05 and DQ1β*02 was observed when we transfected pDQA105Δ (Figure 2A,B).

To assess if the overexpression of surface molecules corresponded to the increment of transcripts, we performed an absolute quantification of DQA1 and DQB1 mRNA by qPCR, using allele-specific primers (Table 2) at 48 h after nucleofection. In Figure 2C, we showed the copy number of transcripts expressed by non-transfected DR1/DR3 B-LCL#5. As already reported in [8], at a steady-state, the amount of endogenous DQA1*05 and DQB1*02 CD-associated transcripts was 5-fold higher, in comparison to the amount of non-CD associated DQA1*01 and DQB1*05 mRNA. Following the nucleofection of DR1/DR3 B-LCL#5 with pDQA105, we observed a 4.7-fold increase of DQA1*05 mRNA, including ectopic and endogenous transcripts, and 5.5-fold increase of DQB1*02 endogenous mRNA (Figure 2D). A slight increase has been quantified also for non–T1D associated transcripts, DQA1*01 (2,3 fold) and DQB1*05 (1,8 fold) mRNA, since they are not significant (Figure 2D). When we transfected pDQA105Δ, the cDNA deleted of 3’UTR, we did not observe any mRNA increase. Analogously, following the transfection of pDQB102, we observed an approximate 3-fold increase in DQA1*05 mRNA and endogenous DQB1*02 mRNA (Figure 2D), while the other transcripts showed only a low increase.

**Figure 3:**
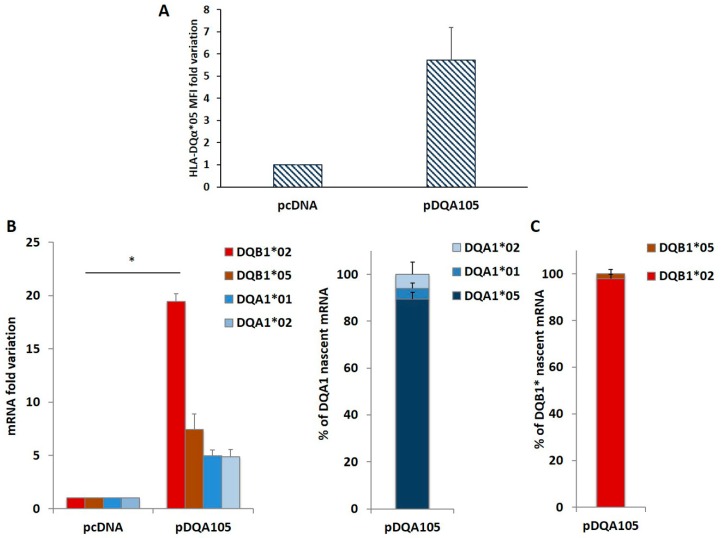
Coregulated expression of DQA1*05 and DQB1*02 CD risk alleles in DR7/DR14 B-LCL and nascent RNA. This cell line was transfected with pcDNA and pDQA105 constructs. In panel **A** we show the MFI assessed by flow cytometry. Panel **B** reports the fold variation of endogenous DQA1*01, DQA1*02, DQB1*02 and DQB1*05 mRNA. P value was calculated respect to cells transfected with empty vector (* *p* < 0.05). Panel **C** shows the quantity of nascent endogenous mRNA following pDQA105 transfection. The left histogram shows the ectopic DQA1*05 mRNA in addition to the endogenous DQA1*01 and DQA1*02 mRNA, as percentage of total DQA1 transcript. The right histogram reported the amount of nascent endogenous DQB1*02 and DQB1*05 mRNA as percentage of total DQB1 transcript.

To confirm this phenomenon, we repeated the same transfection in DR7/DR14 B-LCL lacking the DQA1*05 allele. The ectopic expression of the pDQA105 construct induced the synthesis of DQα1*05 surface molecule (Figure 3A) and DQA1*05 mRNA (Figure 3B). Notably, we appreciated a 19.5-fold increase in the endogenous DQB1*02 mRNA (Figure 3B). Although in a reduced amount, the DQA1*01, DQA1*02, and DQB1*05 transcripts increased (5-, 4.9-, and 7.4-fold, respectively, Figure 3B). These results demonstrated that the ectopic expression of a full-length DQA1*05 cDNA induced a marked rise of DQB1*02 mRNA expression.

We investigated if the overexpression of endogenous mRNA, following the nucleofection is determined by *de novo* transcription. We treated DR7/DR14 B-LCL with the 5-ethynyluridine (EU) that is incorporated into the nascent RNA. New synthetized EU-labeled RNA, is quantified by cDNA preparation and qPCR. The results are reported in Figure 3C as the percentage of each transcript on the 100% total mRNA. We observed that the high expression of DQA1*05 mRNA with respect to DQA1*01 and DQA1*02 is mainly represented by newly synthetized mRNA (left histogram) and this transcript induced *de novo* transcription of endogenous DQB1*02 (Figure 3C, right histogram). The amount of DQA1*01, DQA1*02, and DQB1*05 mRNA was unaffected.

### 3.3. DR7/DR14 B-LCLs Transfected with pDQA105 Efficiently Stimulated Gluten-Specific CD4^+^ T Cells

**Figure 4:**
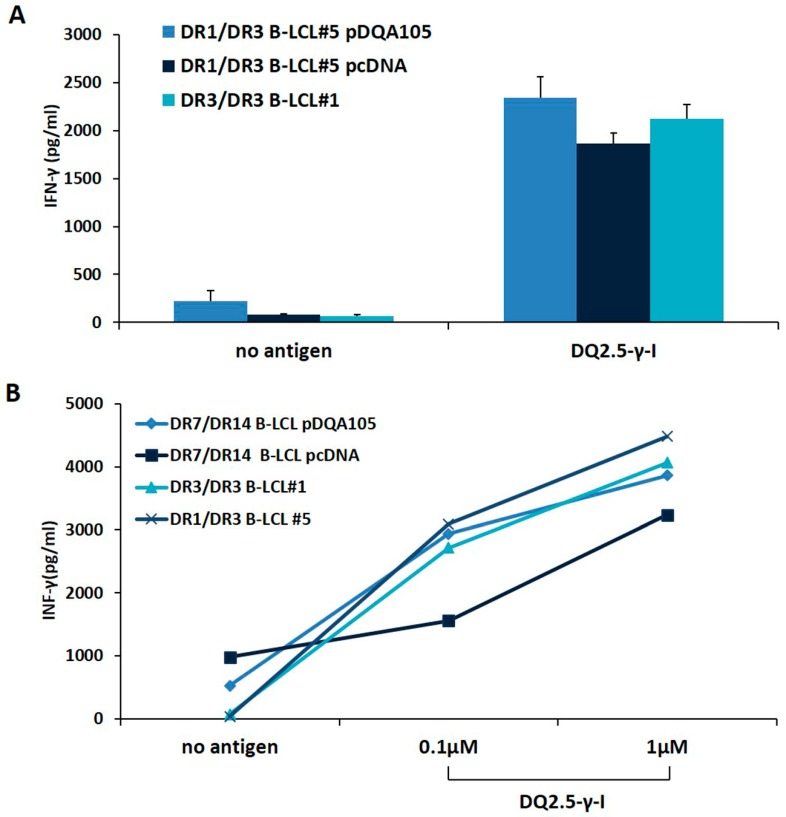
The antigen presenting properties of B-LCLs transfected with pDQA105. The antigen presenting capability of B-LCL pulsed with immunodominant DQ2.5-γ1 gliadin peptide were measured by assessing the activation of intestinal CD4^+^ T cell line. Panel **A** shows the IFN-γ production by CD4 stimulated by DR3/DR3 B-LCL#1 and DR1/DR3 B-LCL#5 transfected with pcDNA or pDQA105 constructs. Panel **B** shows the IFN-γ production by CD4 stimulated with DR3/DR3 B-LCL#1, DR1/DR3 B-LCL#5, and DR7/DR14 B-LCL transfected with pcDNA or pDQA105 constructs.

We investigated whether the new synthesized DQ2.5 heterodimer, expressed following APC transfection, was able to stimulate a CD4^+^ T-cell line, previously established from the intestinal biopsies of a homozygous DR3/DR3 CD patient [14]. We stimulated T cells with autologous DR3/DR3 B-LCL#1 or with DR1/DR3 B-LCL#5 transfected with pcDNA or pDQA105 constructs, pulsed with γ-peptide. After 48 h, we measured the INF-γ production on supernatant by ELISA. We found that CD4 T cells, in response to DR1/DR3 B-LCL#5 transfected with pDQA105, produced a similar amount of INF-γ (2340 pg/mL), with respect to cells transfected by pcDNA and compared to autologous DR3/DR3 B-LCL#1 (mean INF-γ: 2125 pg/mL and 1864 pg/mL, respectively, Figure 4A). Overall, these results demonstrated that heterozygous APC, overexpressing full-length DQA1*05 cDNA, retained the same ability to present gluten peptides and stimulate a T-cell response than homozygous APC. We next evaluated the antigen presenting properties of DR7/DR14 B-LCL after transfection with DQA1*05 gene lacking in that cell line and we assessed the activation of the CD4^+^ T cells specific for DQ2.5-γ-1 peptide at 0.1 and 1 µM suboptimal concentrations. We found that the INF-γ induced by DR7/DR14 B-LCL transfected with pDQA105 (2939.7 pg/mL at 0.1, and 3859.3 pg/mL at 1 µM) are not dissimilar to those produced in response to homozygous DR3/DR3 B-LCL #1 (2718.3 pg/mL at 0.1 and 4063.5 pg/mL at 1 µM) at both concentrations (Figure 4B). Similar results have been obtained when T cells were stimulated with DR1/DR3 B-LCL#1 (3089.5 pg/mL at 0.1 µM, and 4482,75 pg/mL at 1 µM). Conversely, a low cytokine production has been detected after stimulation of T cells with DR7/DR14 B-LCL transfected with empty vector (1556.9 pg/mL at 0.1 µM and 3233.98 pg/mL 1 µM), since the DQ2.2 molecule, expressed by this APC, is able to present the gliadin antigenic peptide with low affinity [14,15]. Overall, these data demonstrated that APC lacking the DQ2.5 molecule, presented antigenic gliadin peptide and became capable of stimulating the gliadin-specific CD4 T cells if an ectopic expression of DQA1*05 alleles is induced.

## 4. Discussion

Although the autoimmune diseases, such as celiac disease and type 1 diabetes, are polygenic disorders, the main genetic risk factor is represented by HLA class II genes. The encoded HLA molecules expressed on the surface of APC have a key role in presenting the self-antigens to inflammatory CD4^+^ T lymphocytes. A number of experimental evidences demonstrated that the expression of these molecules, irrespective of the nature of the antigen, influences the achievement of the HLA-peptide complex threshold, needed for activation and proliferation of autoreactive CD4^+^ T cells [16,17]. We have previously demonstrated that DQA1*05 and DQB1*02 alleles, predisposing to CD, when carried by heterozygous APC, are differentially expressed. This marked expression of CD associated alleles in the APC of heterozygous patients resulted in the production of high concentration of DQ2.5 molecules, comparable to those produced in DQ2.5 homozygous cells. As a consequence, the strength of the gliadin-specific CD4^+^ T cells response is mainly dependent on the antigen dose [8]. In the present work, we investigated HLA expression in B-LCL and PBMC from patients affected by T1D and in patients with co-occurrence of T1D and CD. Most heterozygous subjects carried DQA1*03 and DQB1*03 alleles encoding DQ8 molecules, a smaller group presented DQA1*05 and DQB1*02 alleles translating in DQ2.5 heterodimer and the third group displayed all four alleles encoding DQ2.5/DQ8 molecules (Table 1). The results of mRNA quantization confirmed in T1D the phenomenon already demonstrated in CD. Either DQA1*03 and DQA1*05 risk alleles show an expression higher than 50% when in heterozygous with another DQA1 allele non-associated to T1D while, if the two risk alleles are present in the same genotype, we observed a comparable expression. The high expression was revealed also for DQB1*03 allele in LD with DQA1*03 and for DQB1*02 in LD with DQA1*05, in terms of relative percentage.

Notably, in DQ2.5/DQ8 positive APC that carry the two risk haplotypes, we found a comparable amount of DQA1*03 and DQA1*05, as well as that of DQB1*03 and DQB1*02 mRNA. These findings indicate that among the risk alleles there is not an expression hierarchy and their protein products are expressed at same level. The consistency of our observation is fulfilled when DQ2.5 haplotype is not associated to DQ8, as we found that DQA1*05 is more abundant than DQA1*03, and DQB1*02 is more abundant than DQB1*03. In conclusion, our findings suggested that the allele-specific transcripts have to reach a sort of “threshold level” to mediate the self-antigen presentation, and that a precise hierarchy of expression exists between the DQ alleles predisposing, or not, to autoimmunity. In addition, we found that all T1D risk alleles are regulated accordingly to a mechanism responsible for increased gene expression, that guarantees a coordinated mRNA synthesis of DQA1 and DQB1 genes mapped on the same chromosome.

To investigate this mechanism, we used a known T cells model system that allows us to explore also the functional aspect. Following transfection of DQA1*05 cDNA in a B-LCL expressing the DQ2.5 molecule, we observed an increased endogenous amount of DQB1*02 mRNA, reaching the same quantity of DQA1*05 transcripts. Differently, by transfecting pDQA105Δ we did not observe variations, confirming the important role of 3′UTR in the modulation of transcript stability [10]. Following transfection of pDQA105 in the B-LCL not carrying DQA1*05 allele, we observed a rise in the expression of the endogenous DQB1*02 allele (Figure 2). In conclusion, we demonstrated that the expression of the two genes encoding the surface molecule DQ2.5 is coordinated. To investigate whether the DQB1*02 synthesis was due to the de novo transcription, we monitored and quantified the incorporation of EU, the uridine analog labeling the nascent RNA (Figure 3B) and demonstrated that the stoichiometric balance between the two transcripts of risk alleles in LD is determined by a coordinated transcription. When we modified their equilibrium by ectopic DQA1*05 expression, we stimulated the transcription of endogenous DQB1*02 mRNA.

To explain the mRNA coordinate transcription we suggested that the chromatin, through the formation of loop structures, arranged the promoter of alpha allele close to the promoter of beta allele ensuring the coordination of transcription [18]. Moreover, in our previous papers, we demonstrated that the balance of two transcripts was determined by a coordination not only of the transcription but also of the RNA processing. We proposed that UTRs of alpha and beta transcripts are associated, just after their synthesis, with a protein complex that guarantees a coordinate export from the nucleus and a coordinate turnover [10].

Finally, the antigen presentation experiments confirmed that the heterozygous DR1/DR3 B cells retains the same ability to present the gliadin antigen than homozygous DR3/DR3 B cells and that the overexpression of DQA1*05 did not further increase the density of DQ2.5 heterodimer. This result clearly indicated that DQ2.5 expression reaches a “plateau” and the immune response is dependent on the dose of antigen. However in DR7/DR14 B cells expressing DQ2.2 heterodimer the ectopic expression of DQA1*05 gene induces the new synthesis of DQ2.5 heterodimer, which is able to present gluten antigen and to stimulate gliadin-specific CD4^+^ T cells.

In conclusion, we demonstrated that the two alleles in LD, DQA1*05, and DQB1*02, encoding the DQ2.5 molecule, and DQA1*03 and DQB1*03, encoding the DQ8 molecule, showed a balanced expression. A coordinated transcription is probably ensured by the chromatin structure while the binding of the protein complex with the UTRs regulated their balanced turnover [8].

Our overall findings demonstrated that, in addition to predisposing genotype, the high expression of risk alleles is relevant in the establishment of autoimmunity. In fact, the magnitude of the T cell activation and proliferation is related not only to the nature of epitopes but also to the amount of the antigen-HLA class II complexes expressed on the surface of APC.

## Figures and Tables

**Table 1:** HLA-DQ and HLA-DR genotype and phenotype of T1D patients enrolled in the study.

Code	Cells ^a^	Diagnosis	DQA1 Genotype	DQB1 Genotype	DQ Phenotype	DR Phenotype
**§1**	B-LCL	T1D	*03/*05	*02/*03	DQ2.5/DQ8	DR3/DR4
**§2**	B-LCL	T1D	*03/*05	*02/*02	DQ2.5/DQ2.3	DR3/DR9
**§3**	B-LCL	T1D	*05/*05	*02/*02	DQ2.5/DQ2.5	DR3/DR3
**§4**	B-LCL	T1D with CD	*03/*05	*02/*03	DQ2.5/DQ8	DR3/DR4
**§5**	B-LCL	T1D with CD	*01/*03	*05/*03	DQ8/DQ5	DR4/DR16
**§6**	B-LCL	T1D with CD	*01/*05	*02/*05	DQ2.5/DQ5	DR1/DR3
**§7**	B-LCL	T1D with CD	*02/*05	*02/*02	DQ2.5/DQ2.2	DR3/DR7
**§8**	B-LCL	T1D with CD	*05/*05	*02/*02	DQ2.5/DQ2.5	DR3/DR3
**§9**	PBMC	T1D	*01/*05	*02/*05	DQ2.5/DQ5	DR1/DR3
**§10**	PBMC	T1D	*01/*05	*02/*06	DQ2.5/DQ6	DR3/DR15
**§11**	PBMC	T1D	*01/*05	*02/*05	DQ2.5/DQ5	DR1/DR3
**§12**	PBMC	T1D	*01/*03	*03/*05	DQ8/DQ5	DR4/DR1
**§13**	PBMC	T1D	*03/*05	*02/*03	DQ2.5/DQ8	DR3/DR4
**§14**	PBMC	T1D	*03/*05	*02/*03	DQ2.5/DQ8	DR3/DR4
**§15**	PBMC	T1D	*01/*03	*03/*05	DQ8/DQ5	DR4/DR1
**§16**	PBMC	T1D	*05/*05	*02/*03	DQ2.5/DQ7	DR3/DR5
**§17**	PBMC	T1D	*01/*05	*02/*05	DQ2.5/DQ5	DR3/DR1
**§18**	PBMC	T1D	*03/*03	*03/*03	DQ8/DQ8	DR4/DR4
**§19**	PBMC	T1D	*01/*03	*03/*05	DQ8/DQ5	DR4/DR1
**§20**	PBMC	T1D	*03/*03	*03/*03	DQ8/DQ8	DR4/DR4
**§21**	PBMC	T1D	*01/*03	*03/*05	DQ8/DQ5	DR4/DR1

^a^ the immune cell source for HLA genotyping.

**Table 2:** Primers used for qPCR.

*Gene*	*Primers*	*Sequences 5′ → 3′*
**β-Actin**	ACT-F ACT-R	TCATGAAGTGTGACGTTGACA CCTAGAAGCATTTGCGGTGCAC
HLA-DQA1*01	DQA1*01-F DQA1*R	CGGTGGCCTGAGTTCAGCAA GGAGACTTGGAAAACACTGTGACC
HLA-DQA1*02	DQA1*02-F DQA1*R	AAGTTGCCTCTGTTCCACAGAC GGAGACTTGGAAAACACTGTGACC
HLA-DQA1*03	DQA1*03-F DQA1*R	CTCTGTTCCGCAGATTTAGAAGA GGAGACTTGGAAAACACTGTGACC
HLA-DQA1*05	DQA1*05-F DQA1*R	CTCTGTTCCGCAGATTTAGAAGA GGAGACTTGGAAAACACTGTGACC
HLA-DQB1*02	DQB1*02-F DQB1*R	TCTTGTGAGCAGAAGCATCT CAGGATCTGGAAGGTCCAGT
HLA-DQB1*03	DQB1*03-F DQB1*R	CGGAGTTGGACACGGTGTGC CAGGATCTGGAAGGTCCAGT
HLA-DQB1*05	DQB1*05 DQB1*R	ACAACTACGAGGTGGCGTACC CAGGATCTGGAAGGTCCAGT
HLA-DQB1*06	DQB1*06-F DQB1*R	CAGATCAAAGTCCGGTGGTTTC CAGGATCTGGAAGGTCCAGT

**Table 3:** DQA1 and DQB1 mRNA fold change in different APC.

Cells	Patients	mRNA Alleles	Fold Change	Cells	Patients	mRNA Alleles	Fold Change
B-LCL	**§1**	DQA1*05 = DQA1*03	1	B-LCL	**§5**	DQB1*03 > DQB1*05	3.2
	**§4**						
B-LCL	**§2**	DQA1*05 > DQA1*03	2.7	B-LCL	**§6**	DQB1*02 > DQB1*05	4.6
B-LCL	**§5**	DQA1*03 > DQA1*01	3.2	B-LCL	**§1**	DQB1*02 = DQB1*03	1
					**§4**		
B-LCL	**§6**	DQA1*05 > DQA1*01	3.0	PBMC	**§9**	DQB1*02 > DQB1*05	5.6
					**§11**		
					**§17**		
B-LCL	**§7**	DQA1*05 > DQA1*02	2.7	PBMC	**§10**	DQB1*02 > DQB1*06	5.9
PBMC	**§9**	DQA1*05 > DQA1*01	4.8	PBMC	**§19**	DQB1*03 > DQB1*05	3.1
	**§10**				**§21**		
	**§11**				**§12**		
	**§17**				**§15**		
PBMC	**§12**	DQA1*03 > DQA1*01	2.9	PBMC	**§13**	DQB1*02 = DQB1*03	1
	**§15**						
	**§19**				**§14**		
	**§21**						
PBMC	**§13**	DQA1*05 = DQA1*03	1	PBMC	**§16**	DQB1*02 > DQB1*03	3.3
	**§14**						
